# Metabolic Contributions to Pathobiology of Asthma

**DOI:** 10.3390/metabo13020212

**Published:** 2023-01-31

**Authors:** Tamanna Roshan Lal, Laura Reck Cechinel, Robert Freishtat, Deepa Rastogi

**Affiliations:** 1 Rare Disease Institute, Children’s National Hospital, Washington, DC 20012, USA; 2 Departments of Pediatrics and Genomics and Precision Medicine, George Washington University School of Medicine and Health Sciences, Washington, DC 20052, USA

**Keywords:** asthma, metabolomic, macrometabolic, micrometabolic

## Abstract

Asthma is a heterogenous disorder driven by inflammatory mechanisms that result in multiple phenotypes. Given the complex nature of this condition, metabolomics is being used to delineate the pathobiology of asthma. Metabolomics is the study of metabolites in biology, which includes biofluids, cells, and tissues. These metabolites have a vital role in a disease as they contribute to the pathogenesis of said condition. This review describes how macrometabolic and micrometabolic studies pertaining to these metabolites have contributed to our current understanding of asthma, as well as its many phenotypes. One of the main phenotypes this review will discuss in further detail is obesity as well as diabetes. Distinct roles of metabolites in endotyping asthma and their translation to potential therapy development for asthma is also discussed in this review.

## 1. Introduction

Traditionally, asthma was thought to be a single entity described as bronchial hyperactivity with atopy and characterized by high peripheral and airway eosinophilic inflammation [1,2,3]. However, over the past two decades, there is increased awareness that asthma is a heterogenous disorder. It includes several distinct subtypes, including varying phenotypes (e.g., young atopic, obese middle-aged, and elderly), some of which can be distinguished by differences in biomarkers (i.e., endotypes) [2]. With the continued increase in childhood obesity, one phenotype that is garnering a lot of interest is the “obese asthma” phenotype [4,5]. Studies show that obesity is a risk factor for asthma, which is more severe than healthy-weight asthma, with higher need for hospitalization in the intensive care unit, greater pulmonary function deficits, and decreased responsiveness to existent management options [6,7]. However, the distinguishing features and the biomarkers for disease is not well understood. In this review, we discuss the existing literature on obesity-related asthma, with a focus on the role of metabolic abnormalities and identify areas of future research.

The existing literature highlights the vital role of metabolomics. It is an important emerging method of understanding phenotypes of complex disorders such as obesity-related asthma by distinguishing metabolic patterns to facilitate a better understanding of disease phenotype and, thereby, the development of the personalized management of asthma. The emerging studies of metabolomics in asthma are defining various phenotypes of this complex disorder as well as delineating the pathogenesis of disease from a metabolic perspective [8]. The current literature suggests that metabolic derangements are routinely present in patients with the “obese asthma” phenotype. The metabolic derangements overlap with those present in metabolic syndrome, which includes five categories: abdominal obesity, hypertriglyceridemia, reduced high-density lipoprotein (HDL) levels, hypertension, and insulin resistance/hyperglycemia [9]. While we continue to further understand the etiology of metabolic syndrome, there are data that show it is associated with worsening lung function [10] by modulating airway responses in asthma [11]. For example, neuropeptide glucagon-like peptide-1 (GLP-1) and nitric oxide (NO) signaling pathways are shown to be dysregulated in metabolic syndrome [12].

Given these early findings in the metabolomics of asthma, this review will discuss these updates, categorized as the contribution of macrometabolic and micrometabolic measures to asthma. The macrometabolic section will discuss the known association of carbohydrates and lipids, with more detail on obesity-related asthma. The diabetes phenotype associated with asthma is discussed in this section. Micrometabolic association in asthma will look at the relationship of sphingolipids, amino acids, short chain fatty acids (SCFA), and vitamins. The goal of this review is to provide an update on the distinct roles for these metabolites in endotyping asthma as well as its translation to therapy development for this highly heterogenous disease.

## 2. Macrometabolic Associations in Asthma

### 2.1. Altered Carbohydrate Metabolism Is Associated with Disease Burden in Obesity-Related Asthma

**Figure 1:**
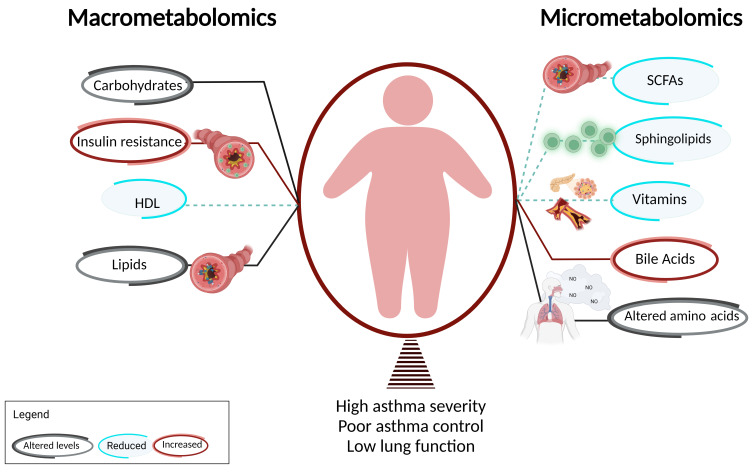
Macrometabolites, micrometabolites, and obesity-related asthma. This figure summarizes the association of obesity-related asthma with macrometabolites and micrometabolites and their underlying mechanisms. T cells are shown in green and FFA receptors are marked in blue.

Metabolic dysregulation, including insulin resistance (IR) and altered glucose metabolism, have been consistently associated with childhood and adult asthma. IR is a well-described obesity-mediated metabolic complication that has been linked with obesity-related asthma [13,14,15]. There is a correlation between obesity-mediated inflammation and IR, which likely contributes to asthma in obese children [16,17]. Children with asthma are prone to have higher prevalence of IR compared to patients without asthma [18,19,20] as quantified by acanthosis nigricans [18,19,20]. There is also evidence of an inverse relationship between IR and pulmonary function [11]. Higher levels of IR are associated with a lower FEV_1_/FVC ratio, the ratio of the forced expiratory volume in the first one second to the forced vital capacity of the lungs, which is the most consistently used pulmonary function index for the quantification of airflow obstruction (Figure 1).

In a cross-sectional study of adolescents, the inverse association between obesity and FEV_1_ and FVC values was more pronounced among obese children with IR [21]. A prospective study on a community-based cohort of 4827 Korean adult participants over four years reported a negative linear association between homeostatic measurement of IR (Homeostatic Model Assessment for Insulin Resistance, HOMA-IR) and annual changes in percent predicted FEV1 and FVC, particularly in subjects above 50 years of age, after adjusting for other risk factors, such as smoking, physical activity, and BMI [22]. Direct evidence of the adverse effects of insulin on lung function was supported by a greater than 15% decrease in FEV_1_ in 1.3% of patients with type 1 diabetes and 5% of patients with type 2 diabetes mellitus following administration of inhaled insulin as therapy for diabetes [23]. The FEV_1_ decline resolved within six weeks of the discontinuation of inhaled insulin, even after up to two years of therapy, supporting the reversibility of the effect of insulin on lung function.

Interestingly, IR is also an independent predictor of expiratory reserve volume (ERV), when adjusted for general and truncal adiposity [11,24]. IR directly correlates with the Th1/Th2 ratio [13] and mediates the association of the Th1/Th2 ratio with lung function. Impaired glucose metabolism, without overt IR, has also been associated with airway hyperresponsiveness (AHR), independent of BMI [25]. These findings support a role of metabolic abnormalities in deficient pulmonary function in obese asthma, independent of BMI or truncal adiposity [24].

### 2.2. Insulin Resistance Influences Asthma Phenotype Partly via Effects on Airway Smooth Muscle

The pathophysiologic mechanisms by which IR impacts pulmonary physiology have been investigated to some degree. Airway smooth muscle (ASM) cells express insulin receptors and develop a pro-contractile phenotype when exposed to insulin; this effect may be more pronounced in obesity [26,27]. Pharmacological agents that alter insulin and glucose metabolism have been linked with decreased disease burden in obese asthma [28,29,30]. Since obesity-induced IR is associated with compensatory hyperinsulinemia, we speculate that, like muscle, the ASM retains insulin sensitivity and may, therefore, have an augmented response to insulin exposure. However, the direct impact of IR and hyperinsulinemia on lung tissue is poorly understood.

There are few studies on human tissue, but the mechanisms by which IR and hyperinsulinemia affect lung tissue have been investigated to some extent in murine models. A 16-h exposure to insulin in both obese-prone and obese-resistant murine models potentiated bronchoconstriction with ASM contraction induced by vagal stimulation, which was blocked by atropine, supporting a role of muscarinic receptors [31]. The pro-contractile effect occurred as early as 30 min after insulin exposure. This effect was seen in rat tracheal smooth muscle, which was eventually replicated in human ASM [31]. Validating these findings, Nie et al. showed that AHR to vagal stimulation increased in obese-prone animals on a high fat diet (HFD) compared to those fed a low-fat diet or obese-resistant rats; these effects were independent of body weight and body fat [32]. Suppressing insulin significantly reduced vagal-induced bronchoconstriction in rats on HFD, suggesting that hyperinsulinemia, rather than obesity alone, underlie obesity-induced bronchoconstriction via parasympathetic nerves. Similar findings have been reported with bovine ASM [33,34]. Further, since AHR is a balance between muscarinic and adrenergic receptor activation, Xu et al. investigated insulin-mediated effects on β2 adrenergic receptors (β2AR) and found a novel role for transactivation of a G protein-coupled receptor kinase 2 (GRK2)-dependent β2AR-Gi-ERK1/2 cascade in ASM cells that was associated with reduced cAMP accumulation with impaired ASM relaxation [35]. Together, these studies demonstrate that effects of insulin on AHR are mediated by dysfunction of both the M2 muscarinic receptor and the β2AR. Highlighting species-specific differences in ASM responsiveness to insulin, a single study that investigated ASM responses from obese individuals found that a 24-h insulin treatment of ASM was not associated with increased calcium release in response to carbachol [27]. While differences in the duration and dose of insulin exposure may potentially explain these differences, these incongruent results identify a need for further investigation, specifically on human ASM.

In addition to AHR, hyperinsulinemia has been associated with increased α-smooth muscle actin and β-catenin in lung tissue, suggesting proliferation of ASM cells. These effects decreased in response to PI3K-inhibition [36]. These observations were validated in human ASM cells, wherein increasing concentrations of insulin for 72 h was associated with an eightfold increase in cells, and deposition of collagen, which is involved in airway remodeling [36]. This study was the first to define potentially irreversible pro-constrictive and profibrotic effects of prolonged insulin exposure on ASM mediated by PI3/Akt pathway activation. In keeping with these effects of insulin on ASM, treatment with anti-diabetic medications, including metformin, a biguanide, and albiglutide, a glucagon-like peptide-1 receptor (GLP-1R) agonist, have been associated with decreased asthma disease burden [28,29]. However, pioglitazone, a thiazolidinedione, also used as an anti-diabetic medication, does not decrease asthma burden [37]. These discrepant results may be due to differences in mechanisms of action of these anti-diabetic medications. For instance, murine studies on the effects of insulin have reported the altered activation of molecules such as the insulin receptor substrate (IRS-1) and AKT downstream of insulin through altered glycation and nitration, which is due to enhanced oxidative stress [38]. Based on this, glycation modulation by GLP-1R agonists [39] and decrease in oxidative stress by metformin [40], may explain their association with improved asthma control in individuals with obesity and asthma [30]. Metformin also decreases airway inflammation and airway remodeling by modulating 5′-adenosine monophosphate-activated protein kinase α (AMPK-α) activity [41,42]. Pioglitazone, a thiazolidinedione, influences insulin sensitivity via modified fatty acid metabolism. Although metformin is a standard therapy for prediabetes and GLP-1R agonist use is increasing in children [43,44], their effectiveness in childhood obesity-related asthma is not known. Given their effectiveness in decreasing asthma burden in adults and known safety profile in children, we propose that these medication classes may serve as potential novel therapy for childhood obesity-related asthma.

### 2.3. Dyslipidemia and Dysregulation of Fatty Acids Are Associated with Obesity-Related Asthma

Children with asthma are prone to have higher prevalence of dyslipidemia compared to patients without asthma [18,19,20] as quantified by decreased high-density lipoprotein (HDL) in the context of increased low-density lipoprotein (LDL), total cholesterol levels, and triglycerides [18,19,20] (Figure 1). There is also evidence of an inverse relationship between dyslipidemia with pulmonary function [11]. Lower levels of HDL are associated with lower FEV_1_/FVC ratio. Serum HDL is negatively associated with the Th1/Th2 ratio and patrolling monocytes, which are elevated in obesity [13]. In addition to lipoproteins, free fatty acids (FFA), classified based on the length of their carbon chains as small-chain, medium-chain, or long-chain, influence the pathogenesis of metabolic diseases, including obesity, type II diabetes, and atherosclerosis, and have been linked with respiratory diseases, including asthma. Medium-chain FFA (MCFAs) and long-chain FFA (LCFAs) are derived through de novo synthesis or fat intake [45], while small-chain FFA (SCFAs) are synthesized by gut microbiota through fermentation of undigested carbohydrates in the cecum and colon [46]. SCFAs are discussed in greater detail under the micrometabolic section of this review. LCFAs are chronically elevated in obese individuals due to increased adipose tissue [47]. Several G-protein-coupled receptors (GPCRs) function as specific receptors for FFAs (FFARs) such that LCFAs and MCFAs function as ligands for FFAR1 (GPR40) and FFAR4 (GPR120), while SCFAs act as ligands for FFAR2 (GPR43) and FFAR3 (GP141). FFAR expression have been found on airway structural cells including ASM cells. While FFAR1 has been investigated, less is known about the effects of FFAR2, 3, and 4 in response to FFAs. Therefore, there is a need to define the contribution of FFA and FFARs in the pathogenesis of obesity-related asthma.

### 2.4. Dyslipidemia Influences Asthma Phenotype Partly via Its Effects on FFA Receptors

FFAR1 protein is expressed in human ASM cells and bronchial epithelial cells [47], with limited investigation of their role in human ASM function. Activation of FFAR1 by LCFAs or FFAR1/4 agonist (GW9508), caused a sustained increase in acetylcholine-induced contractile tone with a rapid and transient rise in intracellular calcium in human ASM cells in a dose-dependent manner. This was suppressed in FFAR1-knockdown cells compared to the control [47]. While LCFAs additionally attenuated the relaxant effect of isoproterenol, a β2AR agonist, GW9508 did not exert any effect of isoproterenol-induced relaxation, suggesting differences potentially due to the involvement of FFAR1 with or without FFAR4. In addition to increased intracellular calcium, LCFAs and GW9508 induced actin reorganization, which is required for smooth muscle contraction. Collectively, the results suggest that LCFAs activate FFAR1 to induce calcium mobilization in human ASM cells via the Gq-PLC/IP3 pathway. Investigating the effects of FFRA1 activation on ASM proliferation in human and murine models, Matoba et al. additionally reported that LCFA and GW9508 exposure for 48 h was associated with ERK and Akt phosphorylation in human ASM cells, with downstream mTORC1 activation, which induced ASM proliferation [48]. This effect of FFAR1 activation induced ASM proliferation was inhibited by pre-treatment with Gi protein inhibitor (PTX), which abolished both ERK and Akt phosphorylation. Contrasting results were reported by Xu et al., where pre-treatment with TAK875 (another selective FFAR1 agonist) attenuated histamine-induced myosin light chain (MLC) phosphorylation and carbachol-induced MLC phosphorylation, and inhibited ASM shortening in β2AR-desensitized human ASM cells, independent of cAMP levels and PI3/Akt activation [49]. Considering the limited literature, these studies suggest that the differences possibly stem from differences between donors and differences in the FFAR1 agonists, as well as the role of myosin light chain phosphorylation in responsiveness to FFAR receptor engagement by different ligands.

### 2.5. Therapies for Dyslipidemia/FFAs Are Effective in Decreasing Disease Burden of Obesity-Related Asthma

The role of FFAs in causing AHR by modulation of ASM cells, and the effect of statins in lowering FFAs [50], support epidemiological reports of the beneficial effect of statins in the context of asthma [51]. A meta-analysis of randomized controlled trials (RCTs) demonstrated that statins improve Asthma Control Test (ACT) scores and were independently associated with reduction in asthma-related ED visits and hospitalizations [51]. However, the meta-analysis did not find an association of statin use with pre- and post-bronchodilator FEV_1_, and peak expiratory flow (PEF), suggesting that the effect of statins on FFAs may not directly translate into a decrease in AHR. Given the dearth of mechanistic studies of how lipid lowering agents influence asthma disease burden, we speculate that mechanistic differences between lipid lowering agents may underlie their effectiveness in decreasing disease burden without influencing pulmonary function. These hypotheses need to be investigated to elucidate mechanistic effects of lipid lowering medications, which will inform their choice for the management of obesity-related asthma. The recent discovery of anti-inflammatory and immunomodulatory properties of statins beyond their cholesterol-lowering function, has resulted in a novel and innovative research avenue relevant to lung diseases. Experimental mouse models have demonstrated that simvastatin attenuates eosinophilic airway inflammation via inhibition of HMG-CoA; however, AHR and lung compliance variations were mevalonate- and HMG-CoA-independent [52], suggesting that statins may not directly influence AHR, but may influence it via their immune modulation. Similar additional mechanistic studies that explain how lipid lowering agents influence asthma disease burden are needed before they can be considered for therapy for childhood obesity-related asthma.

## 3. Micrometabolic Associations in Asthma

In addition to macrometabolic derangements in obesity-related asthma, a better understanding of micrometabolic derangements in asthma will provide insight for personalized medicine, which include both diagnosis and treatment [53]. Multiple micrometabolomic biomarkers have been identified in association with asthma. However, for the purpose of this review, we will be discussing the association of sphingolipids, SCFA, amino acids, vitamins, and bile acids with asthma.

### 3.1. Sphingolipids

Sphingolipids were first identified in the 19th century by Johann Ludwig Wilhelm Thudichu [54]. Multiple bioactive lipids in this category share a common chemical backbone of sphingosine or one of its derivatives [55]. Sphingolipids play an integral role in cellular function via interactions at the cell membrane. Decreased sphingolipid synthesis has been implicated in increased asthma susceptibility. One of the foremost sphingolipids investigated in the context of asthma is Orosomucoid like 3 *(ORMDL3)*, which encodes an ER-resident transmembrane protein that regulates the activity of serine palmitoyltransferase (SPT), the first and rate-limiting enzyme for sphingolipid biosynthesis in cells. Genome-wide association studies (GWAS) have identified polymorphisms in the ORMLD3 gene, located in chromosome 17q21, associated with increased risk of asthma [55]. Murine studies have shown that the overexpression of ORMDL3 reduces sphingolipid synthesis, resulting in AHR in the absence of inflammation [56]. A study investigated its role in a cohort of children aged from 7 to 8 years with mild disease and relatively normal lung function. They found that the FEV_1_/FVC ratio was lower with increased serum dihydroceramide C18 and ceramide C20 (Figure 1). This cohort of patients were predicted to have asthma persistence at ages from 10 to 11 years [57,58]. In addition to its effects independent of inflammation, *ORMDL3* also influences asthma by modulating T cell function [59].

Guo et al. reported on ceramides and sphingomyelin in children with different asthma endotypes [60]. Serum samples were collected on 51 individuals with asthma and 9 healthy individuals and analyzed for sphingolipid levels. Levels of sphingomyelin (SM), including SM34:2, SM38:1, and SM40:1, were significantly decreased in patients with asthma compared to healthy individuals [60]. The study indicated that SM contributes to asthma and may be a protective factor. Sphingolipids may, therefore, serve potential biomarkers and therapeutic targets in asthma as well as help to delineate non-allergic childhood asthma from allergic childhood asthma, facilitating asthma endotyping [60]. In keeping with this study, liquid chromatography–mass spectrometry plasma metabolomic profiles were performed on more than 500 children, at the ages of 6 months and 6 years [61] from the COPSAC2010 (Copenhagen Prospective Studies on Asthma in Childhood) birth cohort. Focusing on plasma sphingolipids, the study showed that lower concentrations of sphingomyelins at the age of 6 months were associated with an increased risk of developing asthma and wheezing before the age of 3 [61]. For children at the age of 6 years, lower concentrations of key phosphosphingolipids (e.g., sphinganine-1-phosphate) were associated with increased airway resistance [61]. In essence, the findings of this study are seminal in identifying a causal link between decreased levels of sphingolipids and the risk of developing early-onset asthma with increased airway resistance. Both de novo and salvage sphingolipid pathways were associated with these outcomes. The findings were specific for airway resistance measured by plethysmography and were not present for FEV_1_ or bronchial hyperreactivity, which suggest that perturbed sphingolipid metabolism is associated with inflammation, particularly in the smaller airways in young children. Moreover, these studies found lower blood sphingolipids in children with non-allergic asthma compared to the controls, again highlighting their role in both endotyping childhood asthma and serving as therapeutic targets [57].

### 3.2. Short Chain Fatty Acids (SCFAs)

SCFAs, which are metabolized by gut bacteria from otherwise indigestible fiber-rich diets, have been shown to improve diseases in animal models of asthma [62]. The most abundant SCFAs are acetate, propionate, and butyrate. SCFAs have important immune-modulating properties, which includes the induction of T regulatory cell differentiation in mice [63,64,65,66], the reduction in eosinophil trafficking, and the survival [67] and promotion of mucosa antibody production [68]. Multiple studies show that decreased fecal SCFAs in infancy is associated with asthma in later life [69,70] (Figure 1). The metabolism of microbial SCFAs is thought to be relevant in airway physiology, with one study showing an increased predicted capacity for SCFA metabolism in association with asthma [71]. A relatively higher concentration of SCFAs acetic acid during pregnancy is associated with reduced asthma/wheezing with allergic sensitization in offspring [71]. These findings highlight the role of SCFAs in the development of asthma and suggest that SCFA-directed treatment could be an effective preventive strategy.

The mechanisms by which SCFAs influence asthma have been investigated to some extent. Both FFAR2 and FFAR3, which are expressed on sinoepithelial cells [50], pulmonary fibroblasts [51], and human ASM [52] are activated by SCFAs such as acetate, propionate, and butyrate. FFAR2 couples to either Gq or Gi proteins, while FFAR3 exclusively uses Gi protein signaling [45]. In murine models, SCFAs have demonstrated a protective effect in allergic airway inflammation mediated by FFAR3 activation [53], while FFAR2-knockout mice showed exacerbated or unresolving inflammation in models of asthma [54]. Mizuta et al. studied the effects of FFAR2 and 3 in human ASM tone modulation [55]. While there is no expression of FFAR2 in human ASM cells, a 15-min treatment with SCFAs induced FFAR3 activation, which provoked transient intracellular calcium increases through the Giβγ-PLC-IP3 pathway but inhibited cAMP production mediated by Gi protein-coupled FFAR3. In addition, SCFAs enhanced acetylcholine-stimulated calcium mobilization and actin reorganization in human ASM cells via FFAR3 stimulation. Although mRNA encoding for FFAR4 has been found in human ASM cells [47], there is limited data regarding its role in ASM regulation. Previously described research by Mizuta showed that FFAR4-knockdown HASM cells were associated with a smaller suppression of calcium mobilization induced by LCFAs compared to FFAR1-knockdown human ASM cells [47]. In addition, the selective FFAR4 agonist TUG-891 failed to induce stress fiber formation or potentiate acetylcholine-induced ASM contraction [45], further supporting the limited role of FFAR4 in ASM tone modulation or ASM cell proliferation. Gq-coupled FFAR4 activation by its ligands (LCFAs and MCFAs) results in elevation of intracellular calcium, and Gq-coupled FFAR4 downstream signaling is purported to involve the ERK and IP3 pathways, yet the precise cascade is still under investigation [46]. Investigated to a limited extent with regards to their effects on SCFAs, statins function as 3-hydroxymethyl-3-methylglutaryl coenzyme A (HMG-CoA) reductase inhibitors, which block the cholesterol biosynthesis pathway in the mevalonate cascade [72]. The depletion of mevalonate ultimately modifies GTPases including RhoA, which plays a role in ASM contractility as well as proliferation [73]. These few studies highlight a role of SCFAs and their modulation in asthma and identify another area for further investigation.

### 3.3. Amino Acids

Metabolomics studies have been performed to search for the role of amino acids and potential biomarkers of asthma [74]. There is evidence that alterations in circulating metabolic measures play a role in asthma (Figure 1). The L-citrulline metabolism is possibly the most investigated amino acid that appears to play a significant part as an important biomarker [74]. The literature has shown that biosynthesis of L-arginine from L-citrulline is sufficient in healthy individuals but there is decreased L-arginine production in those with asthma. L-arginine is vital for the biosynthesis of nitric oxide (NO), which is a bronchodilator known to prevent the progression of asthma [75]. Imbalance in the L-arginine metabolism leading to nitric oxide deficiency and airway constriction can occur through increased arginase activity or by the accumulation of endogenous nitric oxide synthase inhibitors, such as asymmetric dimethylarginine (ADMA) [75]. ADMA and symmetric dimethylarginine (SDMA) are produced by methylation of arginine residues in proteins by protein methyl transferases, which are liberated during proteolysis [76]. In keeping with its role in smooth muscle constriction in vascular diseases [75], ADMA levels are increased in a mouse model of allergic airways inflammation [77]. Thus, it is possible that ADMA competes with L-arginine for binding to NOS in asthma airways, which would result in decreased NO production. The balance between L-arginine metabolism and the release of endogenous inhibitors of these respective enzymes are likely important in understanding the underlying pathologic mechanisms of asthma. In a recent clinical trial, individuals with asthma were given L-citrulline supplementation. Post-supplementation, they were found to have increased exhaled NO (FeNO) and an improved FEV_1_ [78]. Other studies suggest that supplementation with L-arginine may increase FeNO in patients with asthma [79]. Liao et al. hypothesized that patients with severe asthma on treatment with a low or normal FeNO would have fewer exacerbations with L-arginine supplementation over a 3-month period compared with patients with high FeNO [80]. A single-center randomized clinical trial of L-arginine supplementation was performed, where 50 patients with severe asthma were randomized into two groups—24 in the low FeNO group and 26 in the high FeNo group. Unlike the hypothesis of a protective effect of L-arginine on asthma among individuals with low FeNO, this trial did not identify any significant clinical benefits of L-arginine supplementation in the individuals with severe asthma in this study. This may be attributed to the fact that there are limitations with L-arginine supplementation since it undergoes a rigorous first-pass metabolism in the liver and intestine [70]. A study by de Gouw et al. found only some improvement in FEV_1_ in patients with asthma after 1 week of L-arginine supplementation [73], while a separate study conducted in 15 patients with moderate to severe asthma did not show any increase in FeNO [70]. These studies indicate that L-citrulline supplementation should be looked at instead of L-arginine as the potential way forward in terms of asthma management. The safety of L-citrulline supplementation has been proven in a recent study on its efficacy in adults with obesity-related asthma [69].

### 3.4. Vitamins

Change in dietary habits with higher intake of processed foods relative to fruits and vegetables has been associated with higher asthma incidence and disease burden [74]. These changes in dietary habits have directly influenced levels of micronutrients, including vitamins, in individuals, particularly those with overweight/obesity (Figure 1). A cohort of 143 children, which included 72 obese and 71 normal-weight asthmatics, was studied. The objective was to investigate systemic inflammatory patterns and their association with pulmonary function among these children [75]. The study visit comprised a questionnaire on asthma disease burden, including daytime and nighttime symptom frequency as well as medication use [75]. Anthropometric measurements, pulmonary function testing, and a blood draw including 25-OH vitamin D was also obtained. Results showed that vitamin D deficiency was associated with lower pulmonary function among obese children with asthma, but not among normal-weight asthmatic children. Findings indicated that vitamin D deficiency was a possible predictor of both lower airway obstruction and reduced pulmonary volumes among obese children with asthma, independent of systemic inflammation. Thus, the study team suggested perhaps obesity and vitamin D deficiency together may play a role in asthma morbidity [75].

Similarly, carotenoid levels have also been studied to further understand its association with asthma as well as obesity. In a clinical study, 158 children were recruited with the objective to quantify serum carotenoid and fatty acid concentrations as measures of nutritional status as well as investigate their association with disease burden in obese adolescents with asthma [76]. Findings showed that serum levels of total carotenoids were lowest in obese asthmatic adolescents, lower than both healthy-weight asthmatics and obese controls [76]. Incidentally, an inverse association was observed between serum levels of total carotenoids and insulin resistance, again in obese asthmatic children only. Furthermore, there was a direct positive association between total carotenoids and HDL in both obese study groups, suggesting carotenoids could possibly be protective against metabolic dysregulation in pediatric obesity [76]. These findings support the links between vitamin A and D supplementation and lower asthma disease burden [77]. Dietary fiber has also been linked with improved asthma outcomes, with the main mechanism being its role in the alteration/improvement of the gut microbiome, which is intricately linked with SCFAs detailed above. Together, these studies support the approach of modified dietary intake for pediatric obesity-related asthma with augmentation of the intake of fruits and vegetables that are rich in vitamins as well as dietary fiber, which plays a direct role in the health of the gut microbiome. Whether diet modification is more effective than weight loss approaches in decreasing disease burden needs to be investigated.

### 3.5. Bile Acids

Bile acids comprise metabolomic biomarkers that are currently being actively investigated for their multiple roles in health and disease. Primary bile acids are synthesized from cholesterol and conjugated in the liver and are then secreted via the bile duct into the duodenum [78]. In the intestines, bile acids contribute to the digestion of dietary fat by acting as emulsifiers. In the lower GI tract, most of the bile acids are absorbed back into the circulation and returned to the liver, where they are taken up and re-excreted, also known as enterohepatic circulation. The enterohepatic circulation, thus, prevents the loss of cholesterol-containing moieties in the feces [79]. As well as an emulsifying role, bile acids have been described to have bactericidal properties, which can alter the community structure of the gut microbiome [78]. It also appears to have a signaling role, whereby it binds to two receptors, the farnesoid X receptor (FXR), a nuclear receptor, and to Takeda G protein-coupled receptor 5 (TGR5), a GRP. These signaling pathways are thought to be beneficial against obesity and obesity-related conditions [78]. The anti-inflammatory properties of bile acids are hypothesized to have a role to play in obesity-associated asthma [78,80]. Manni et al. looked at plasma bile acids using liquid chromatography high resolution mass spectrometry [81]. Plasma samples from two cohorts of lean and obese healthy individuals and individuals with asthma were analyzed for a panel of 17 bile acids [81]. Plasma levels of glycocholic acid (GCA) were significantly increased in individuals with asthma compared to healthy subjects. When GCA and glycoursodeoxycholic acid (GUDCA) levels were stratified by BMI, the highest concentrations were detected in individuals with asthma and a BMI > 25. In other words, it was suggested that bile acids were altered by obesity and asthma status (Figure 1). Using a murine model of obese allergic airway disease (AAD), analyses of bile acids were performed. β-muricholic acid (βMCA) and tauro-β-muricholic acid (tβMCA) were increased in obese mice with AAD compared to obese controls [81]. This paper has shown an interesting association between asthma pathogenesis and dysregulated bile acid synthesis/levels in the setting of obesity. However, the literature is still limited, which warrants further studies to better understand if bile acid profiles change in relation to asthma, especially in the setting of obesity.

## 4. Conclusions

The various metabolic pathways discussed in this review appear to be interlaced, in relation to the pathogenesis of asthma. Disturbances to these various metabolic processes have demonstrated a detrimental effect to airway inflammation, remodeling, and even oxidative stress. There still appear to be gaps and limitations to the knowledge we have on this subject. However, it is encouraging that the application of metabolomics is providing better understanding to a complex disease. The hope for the future is that the application of metabolomics can further improve the diagnosis of asthma, better delineate the phenotype, and ultimately provide the means to develop personalized treatment.
